# Emergency Department Slit Lamp Interdisciplinary Training Via Longitudinal Assessment in Medical Practice

**DOI:** 10.5811/westjem.18514

**Published:** 2024-08-16

**Authors:** Samara Hamou, Shayan Ghiaee, Christine Chung, Maureen Lloyd, Kelly Khem, Xiao Chi Zhang

**Affiliations:** * Sidney Kimmel Medical College, Philadelphia, Pennsylvania; † Department of Emergency Medicine, Emory University, Atlanta, Georgia; ‡ Department of Ophthalmology, Wills Eye Hospital, Philadelphia, Pennsylvania; § Department of Emergency Medicine, Thomas Jefferson University, Philadelphia, Pennsylvania

## Abstract

**Introduction:**

Eye emergencies make up nearly 3% of US emergency department (ED) visits. While emergency physicians (EP) should diagnose and treat these ophthalmologic emergencies, many trainees report limited ocular exposure and insufficient training throughout their residency to confidently conduct a thorough slit-lamp exam.

**Methods:**

We created an interdisciplinary, simulation-based mastery learning (SBML) curriculum to teach emergency attending physicians how to operate the slit lamp with multimodal learning methodology at a tertiary academic center. The EPs first demonstrate their initial slit-lamp competency with a 20-item checklist, and they then review the necessary curricular content to pass their independent readiness test before completing their in-person teaching and demonstration session with an ophthalmology attending to demonstrate procedural mastery (minimal passing score >90%).

**Results:**

Fifteen EPs were enrolled; all completed the final exam of the curriculum. The pre- and post-curriculum checklist scores increased by an average of seven points (*P* = .002); 86.7% of EPs felt confident in completing a slit-lamp exam after the curriculum, compared to 20% at the beginning. Five of 15 reported teaching learners within the two-month post-curricular period, ranging from 5–30 students. The hands-on teaching was the most positively reviewed element of the curriculum.

**Conclusion:**

The SBML program successfully trained EPs on performing a comprehensive slit-lamp exam with promising results of downstream education to junior learners. We encourage other institutions to leverage SBML as a teaching modality for procedural-based training and advocate cross-discipline education initiatives.

## INTRODUCTION

**Figure 1.**
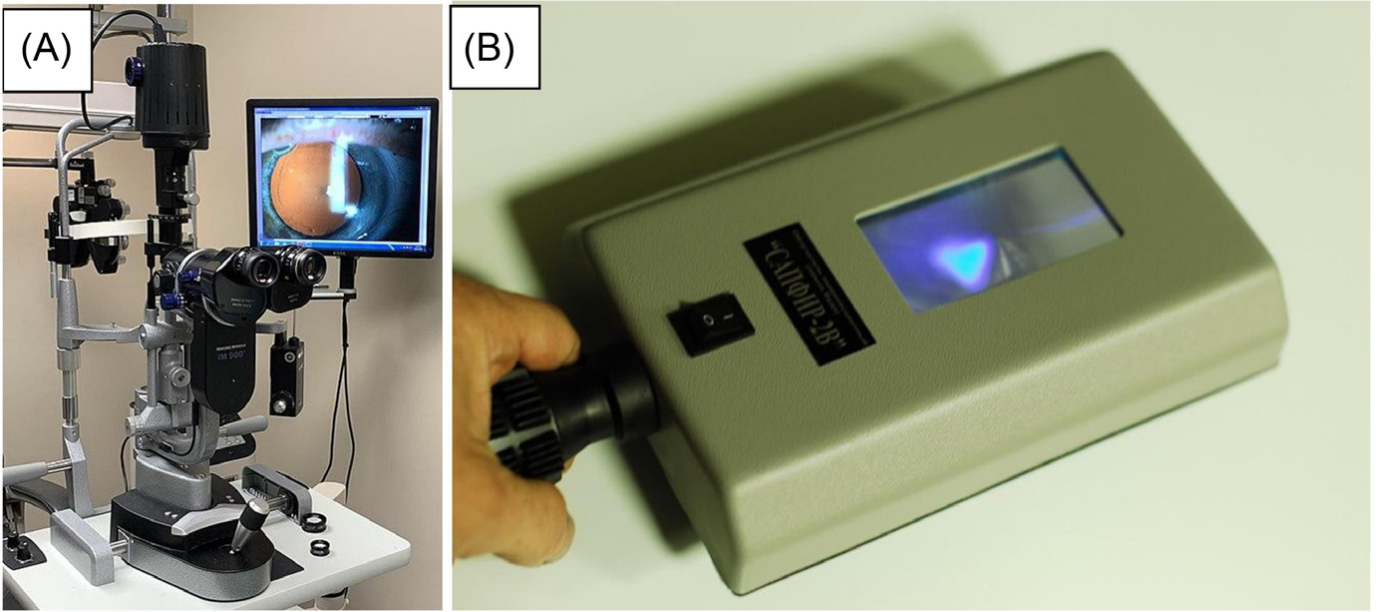
Slit lamp (A) and Wood’s lamp (B).

The slit-lamp^1^ (Figure 1A) is a microscope that allows for a detailed examination of the anterior eye segment using light beam manipulation. The slit-lamp enables physicians to diagnose anterior ophthalmic pathologies such as corneal injuries, iritis, hyphema, hypopyon, and foreign bodies^2^; furthermore, it is essential for performing detailed ophthalmologic exam techniques such as lid eversion, fluorescein examination, and foreign body removal.^3^ The Wood’s lamp^4^ (Figure 1B), in contrast, is a handheld device often used to characterize skin pigmentation, dermal infections, and macroscopic infections with a built-in magnifying lens and ultraviolet (UV) light. The UV capabilities can highlight fluorescein staining during external ocular exams to assess corneal pathologies at lower magnification. While the Wood’s lamp offers a less detailed examination than the slit lamp, it is a more portable diagnostic tool for larger ocular lesions, foreign bodies, or specific reaction to fluorescein staining and meets the needs of the emergency physician (EP) under certain situations.

Eye emergencies make up nearly 3% of US emergency department (ED) visits, the most common of which are traumatic.^5^ ^,^ ^6^ The most common eye injury evaluated in the ED is corneal abrasion (superficial injury to the cornea) and eyelid laceration. Such injuries are best viewed under high-field magnified viewing using the slit lamp to assess for concomitant injuries or co-infections such as corneal ulcers, hypopyon/endophthalmitis, retained foreign body, full thickness corneal laceration, globe ruptures, and seidel testing.^7^ Ocular emergencies such as traumatic globe rupture, ocular foreign body, closed-angle glaucoma, and endophthalmitis are visible only using the slit lamp, and fall within the EP’s scope of practice for diagnosis, triaging, and management.^8^ Mismanaged ophthalmic emergencies can result in inappropriate consultation, excessive testing, financial burden, and even irreversible vision loss.^9^ Despite the significance and frequency of ocular emergencies across the US, many EPs are not confident performing a detailed ophthalmic exam.^10^

Previous literature has found EPs receive fewer than 10 hours of ophthalmic education during residency with low confidence in performing a comprehensive ophthalmic slit-lamp exam.^11^ Ophthalmic education through clerkships and didactics in medical school is also in decline, leading to the unpreparedness of incoming residents before any formal residency training.^11^ ^,^ ^12^ However, it is important that EPs be confident in using the slit lamp to appropriately triage and manage ocular emergencies as part of the Accreditation Council for Graduate Medical Education (ACGME) Emergency Medicine (EM) Milestones Patient Care domain (PC8) – General Approach to Procedures, which designates a set of sequential milestones for overall procedural competency, not focusing on a specific list of procedures.^13^

The optimal learning environment for adult learners to perform a technically challenging procedure should incorporate elements from both the mastery learning model and rapid cycle deliberate practice (RCDP). The mastery learning model ensures that students can master a topic if they receive unlimited time and support in learning and reviewing material until mastery proficiency is reached. Meanwhile, the RCDP model ensures learners can practice skills repetitively while receiving brief, interspersed feedback to achieve a designated proficiency level before proceeding to the next task.^14^ ^,^ ^15^ ^–^ ^17^ Within medical education, simulation-based mastery learning (SBML) models have been successfully implemented across various specialties, such as emergency medicine, general surgery, critical care, and gastroenterology.^18^ ^,^ ^19^ ^,^ ^20^ In light of successful, smaller scaled studies on the effectiveness of slit-lamp training within undergraduate medical education, we propose a SBML procedural training curriculum that can enable adult learners to conduct deliberate performances of intended cognitive or psychomotor skills in sequential order with a repetitive skills assessment.^15^ ^,^ ^21^ ^,^ ^22^ Specific, informative feedback will enable sustained performance improvement to achieve slit-lamp mastery.^23^ Our goal was to design a pilot interdisciplinary course that could teach EPs to complete a comprehensive slit-lamp exam in diagnosing common anterior eye pathology.

## METHODS

Our study, Emergency Department Slit Lamp Interdisciplinary Training via Longitudinal Assessment in Medical Practice (ED SLIT LAMP), is a multicentered, collaborative project that leverages the conceptual frameworks of the mastery learning model and RCDP to ensure proficiency in conducting a comprehensive slit lamp exam. It also serves as a scaffold for deconstructing barriers in traditional siloed medical practices and leads to improved patient care, knowledge synthesis, and resource utilization of our consulting services. The study was conducted at Thomas Jefferson University (TJUH) and the Wills Eye Hospital (WEH) from 2021– 2023. The hospitals with their respective EDs, are 0.2 miles apart, with staff from each institution working as consultants at the other; WEH residents function as ophthalmology consultation for the TJUH ED, while TJUH EPs function as overnight medical emergency consultants at the WEH ED. The geographic and relationship proximity created ideal conditions to develop and pilot a procedural skill competence SBML curriculum.

Emergency physicians were selected as ideal learners due to their level of training and unique teaching responsibilities. Using the TJUH ED listserv we recruited eligible participants and offered staggered financial incentives. For this pilot study, we required a minimum of 12 participants to meet 5% type 1 error and 80% power based on score improvement from baseline testing to post-testing, as referenced by Miller at al.^24^ The ED SLIT LAMP study leveraged talents from content and education experts from both institutions to create an interdisciplinary procedural teaching curriculum. The success of a traditional SBML curriculum is linked to the learners’ skill acquisition. Our study expands this measure to include interdisciplinary collaboration, demonstrating the successful alignment between educational and patient-centered goals that benefit both departments. To evaluate the curriculum, we employed all four levels of the Kirkpatrick model. Using pre- and post-test Likert scale questionnaires, our measurement of success included improved learner confidence (level 1), knowledge acquisition (level 2), willingness of learners to incorporate their skillset in clinical practice (level 3), and dissemination of this knowledge to junior learners (level 4). Any curricular feedback and improvements were extracted for future curricular iterations.

A needs-based analysis conducted at TJUH ED revealed EPs desired hands-on slit-lamp education and training on identifying anterior segment ophthalmic complaints. Since ophthalmology is a recognized component of the American Board of Emergency Medicine exam content, we constructed the pre-test clinical content based on critical and common ocular diagnoses, the most common WEH ED ophthalmology discharge diagnoses, and clinical identifications deemed “can’t miss” by the ED and ophthalmology department.

All curricular contents (lecture materials, video recording, pre-post-post assessments, study surveys, mastery learning checklist) were created by the principal investigator [XCZ] with ophthalmology co-investigators consultation [CC, MEL] based on targeted needs assessment. These materials underwent sequential review by select experts at WEH and were modified sequentially until a consensus was reached. The minimal passing checklist score was determined to be 90%, based on combined determination from ophthalmologist experts at WEH and similar threshold determined by Miller et al.^24^ Each curriculum assessment (Appendix A) was constructed to mirror the natural knowledge, skills, and attitude progression from the ACGME EM Milestones Patient Care Domain (PC8). Due to the multifaceted nature of EM, there is no specific procedural milestone for performing a slit-lamp exam, as described in detail in the ACGME Ophthalmology PC1: Data Acquisition - Basic Ophthalmology Exam and Testing (Level 1).^13^ However, the EM PC8 milestones provide structured language applicable to many ED procedures and advanced device-assisted medical examinations (ie, slit-lamp exam). Please see Table 1 for the correlation between the EM milestone and ED SLIT LAMP assessments.

**Table 1.** Corresponding emergency department slit-lamp assessments to ACGME EM^*^ milestone general approach to procedures.

ACGME EM milestone PC8	Bolded PC8 elements relatable to performing a slit lamp exam	Correlating ED SLIT LAMP assessments
Level 1	Identifies indications for a procedure and pertinent anatomy and physiology. Performs basic therapeutic procedures (eg, suturing, splinting)	Appendix A–Part II (clinical image examination)
Level 2	Assesses indications, risks, benefits, and alternatives and obtains informed consent in low- to moderate-risk situations. Performs and interprets basic procedures, with assistance. Recognizes common complications	Appendix B–Part I (slit lamp technical) Appendix B (final checklist)
Level 3	Assesses indications, risks, and benefits and weighs alternatives in high-risk situations. Performs and interprets advanced procedures, with guidance. Manages common complications	Appendix A–Part III (ophthalmology exam mix-n-match
Level 4	Acts to mitigate modifiable risk factors in high-risk situations. Independently performs and interprets advanced procedures. Independently recognizes and manages complex and uncommon complications	Appendix B (final checklist)
Level 5	Teaches advanced procedures and independently performs rare, time-sensitive procedures. Performs procedural peer review	Appendix C–ED SLIT LAMP surveys

* *ACGME EM*, Accreditation Council for Graduate Medical Education Emergency Medicine; *PC*, patient care; *ED SLIT LAMP*, Emergency Department Slit Lamp Interdisciplinary Training.

The longitudinal curriculum included four unique time points (Time 0–3) of intervention staggered over six months (Appendix A, Appendix B). At Time 0, participants completed an in-person baseline slit-lamp exam that was video-recorded and reviewed by two independent investigators [XCZ] [MEL]. At Time 1, the participants gained access to an asynchronous learning packet that consisted of a PowerPoint presentation on common ED eye complaints, digital library links to the WEH Manual, slit-lamp checklist, and a video recording of a comprehensive slit-lamp examination.^25^ The participants also gained access to an independent readiness assessment (IRAT), which was required to be completed within 30 days with a minimum score of 90% before proceeding to the next in-person phase of the study (Appendix A).

Upon achieving the passing IRAT score, they were invited to participate in the Time 2 (in-person) SBML portion of the study where they were to complete an in-person demonstration of a comprehensive slit-lamp exam by a board-certified ophthalmologist [CC] on a standardized patient volunteer. Following the demonstration, participants were given unlimited time for RCDP with brief, interspersed feedback under the observation and teaching from the ophthalmologist. Participants were required to complete a minimum 18 of 20 checklist items to achieve mastery (Appendix B). Upon completing the final checklist, the participants were asked to complete a course evaluation and learner confidence survey (Appendix C) with Likert scaling, subjective commentary, and a validated 5-item Critical Incidence Questionnaire (CIQ) for curricular improvement. Given the unpredictability nature of the “unlimited attempts” at Time 2, all participants were scheduled at two-hour intervals to allow for device preparation, one to two re-attempts, debriefing, survey completion, and general troubleshooting. At Time 3, participants completed a 60-day post-examination survey, assessing their ocular knowledge, slit-lamp confidence, clinical teaching opportunities, and relevant interprofessional relationships.

We used a Wilcoxon signed-rank test to differentiate the checklist scores between the curricular intervention by incorporating collected paired data before and after the training, median and interquartile range values of subtotal scores at two-time points.^26^ We used McNemar’s test to comparing each categorical sub-score (Yes/No) by time points and corresponding *P*-value within the same population.^27^ The descriptive summaries of survey questions at Time 0, Time 2, and three-month follow-up were analyzed using Bonferroni adjusted *P*-values (multiplying *P*-value from Wilcoxon signed-rank test by the number of multiple tests, doubling the *P*-values), which was directly compared to the pre-specified 5% significance level. All statistical analyses were performed using R 4.1.2 (R Foundation for Statistical Computing, Vienna, Austria).^28^

This study was approved by the institutional review board at Thomas Jefferson University Hospital (TJUH) in Philadelphia, PA. Informed consent was obtained from participating physicians. This study was funded by the Center for Faculty Development and Nexus Learning Pedagogy Grant at Thomas Jefferson University.

## RESULTS

Fifteen EPs (six females and nine males) were enrolled in ED SLIT LAMP during the two-year period; none were lost to follow-up. All participants were board-certified EPs with an average clinical experience of 7.8 years post-residency graduation. All EPs completed the final exam of the curriculum in one attempt and all under 60 minutes.

Table 2 lists the 20 steps of the slit-lamp exam curriculum checklist, comparing participant results from recorded slit-lamp attempts (Time 0) to the final in-person assessment (Time 2). The intra-class correlation in test scores between EPs and ophthalmologists at Time 0 (2 raters) was 0.98. We found a significant increase between the checklist scores before and after the education initiative, 12.0 to 19.0, *P* = 0.002.

**Table 2.** Descriptive summary of checklist evaluation at pre- and post-curricular and comparison between time points.

Checklist item	Performed	Time 0, N(%) (N = 15)	Time 2, N(%) (N = 15)	*P*-value from exact McNemar’s test
1 - Identify slit lamp anatomy.	Yes	13 (86.7%)	15 (100%)	0.50
2 - Apply transparent face shield over the slit lamp (COVID).	Yes	4 (26.7%)	15 (100%)	<0.001
3 - Sanitize forehead and chin rest for the patient.	Yes	5 (33.3%)	14 (93.3%)	0.004
4 - Apply topical tetracaine/proparacaine on patient’s eyes.	Yes	8 (53.3%)	12 (80.0%)	0.22
5 - Unlock instrument base and shift by pulling toward you.	Yes	15 (100%)	15 (100%)	NA
6 - Adjust eye pieces for your interpupillary distance and refractive error.	Yes	10 (66.7%)	14 (93.3%)	0.22
7 - Adjust table height and/or chair(s) - neither patient nor examiner should be hunched over.	Yes	12 (80.0%)	14 (93.3%)	0.50
8 - Instruct patient to close eyes while you power up by turning on the light source at low voltage setting and focus on right eyelid. Position patient in slit lamp with forehead touching the horizontal bar and chin in the chin rest.	Yes	4 (26.7%)	15 (100%)	<0.001
9 - Set magnification on lowest settings (10x to 12x), illumination at largest aperture and widest slit beam.	Yes	12 (80.0%)	15 (100%)	0.25
10 - Adjust chin rest so the patient is sitting comfortably with their chin on the chinrest and their forehead against the headrest.	Yes	12 (80.0%)	15 (100%)	0.25
11 - Practice macro and micro adjustments of the sliding base with joystick.	Yes	14 (93.3%)	15 (100%)	1.00
12 - Adjust microscope 90° to facial plane with illumination set at 45° angle (angle left for patient’s right eye, and right for left eye).	Yes	7 (46.7%)	15 (100%)	0.008
13 - Perform outer structure evaluation.	Yes	14 (93.3%)	15 (100%)	1.00
14 - Perform anterior chamber evaluation.	Yes	5 (33.3%)	15 (100%)	0.002
15 - Look for cells and flare.	Yes	4 (26.7%)	12 (80.0%)	0.02
16 - Place a drop of tetracaine/proparacaine on a sterile fluorescein strip.	Yes	15 (100%)	15 (100%)	NA
17 - Place the fluorescein in the inferior fornix of the eye by pulling down on the lower lid and gently touching the bulbar conjunctiva with the fluorescein strip.	Yes	9 (60.0%)	15 (100%)	0.03
18 - Adjust cobalt blue filter on diaphragm wheel at maximum beam height and medium width slit setting for fluorescein evaluation.	Yes	14 (93.3%)	15 (100%)	1.00
19 - Focus the slit beam at 9:00 position on limbus. Move across the cornea to the 3:00 position by tilting joystick laterally.	Yes	12 (80.0%)	15 (100%)	0.25
20 - Pull instrument base toward you when finished and lock in position. Turn off.	Yes	4 (26.7%)	13 (86.7%)	0.004
		Time 0, median [IQR]	Time 2, median [IQR]	*P*-value from Wilcoxon signed rank test
Subtotal score		12.0 [10, 16]	19.0 [19, 20]	0.002

*IQR*, interquartile range.

The most notable differences between the pre- and post-curricular intervention were as follows: 1) instructing the patient to close their eyes while powering up and positioning the patient in the slit lamp with the forehead touching the horizontal bar and chin in the chinrest (*P* < 0.001); 2) adjusting the microscope 90 degrees to facial plane with illumination set at a 45-degree angle (*P* = 0.008); 3) performing an anterior chamber evaluation (*P* = 0.002); 4) looking for cells and flare (*P* = 0.021); and 5) placing fluorescein in the inferior fornix of the eye (*P* = 0.031). The most missed steps at the baseline exam were: 1) applying a transparent face shield (26.7%); 2) instructing patients to close their eyes when the machine was turned on (26.7%); 3) looking for cells and flare (26.7%).

**Figure 2.**
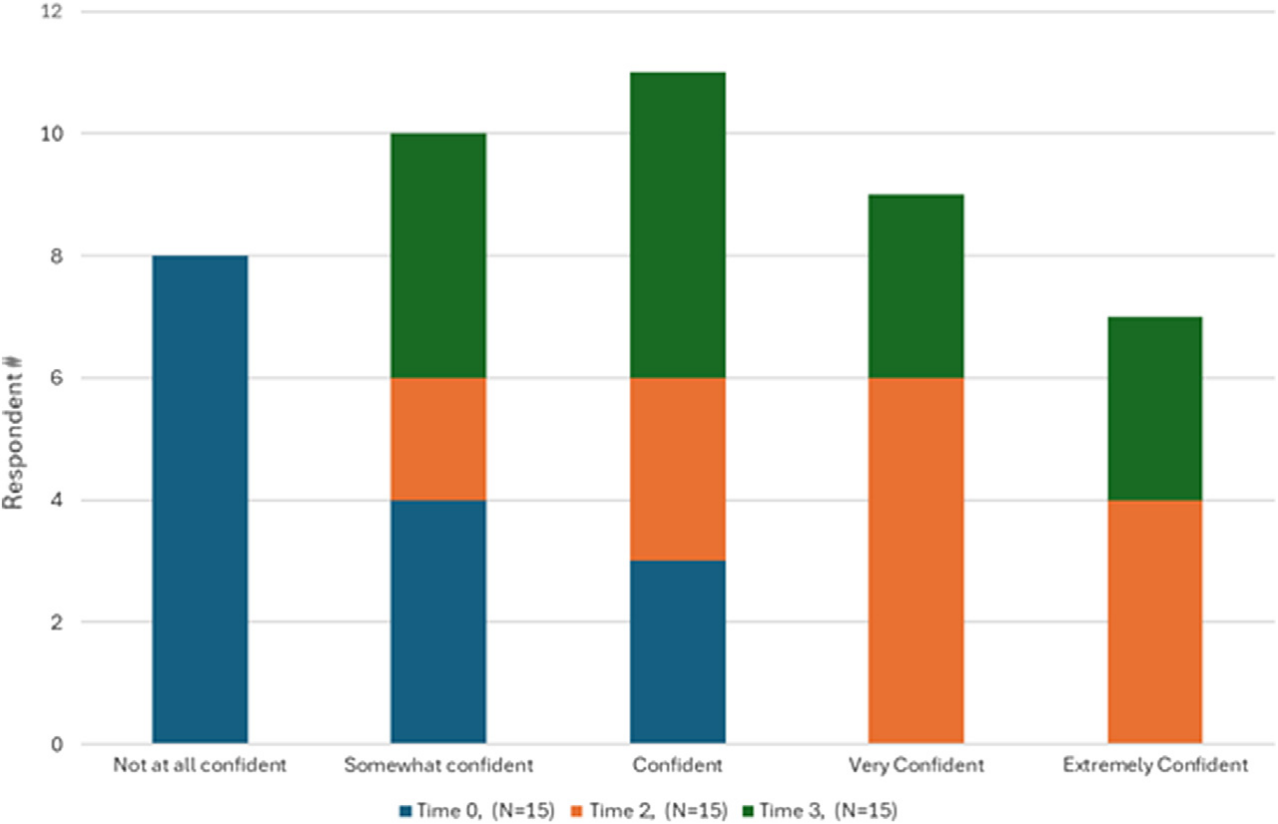
Learner confidence in performing the slit-lamp exam at Time 0 (pre-curricular), Time 2 (immediate post-SBML curriculum), and Time 3 (2-month post-SBML curriculum).

**Figure 3.**
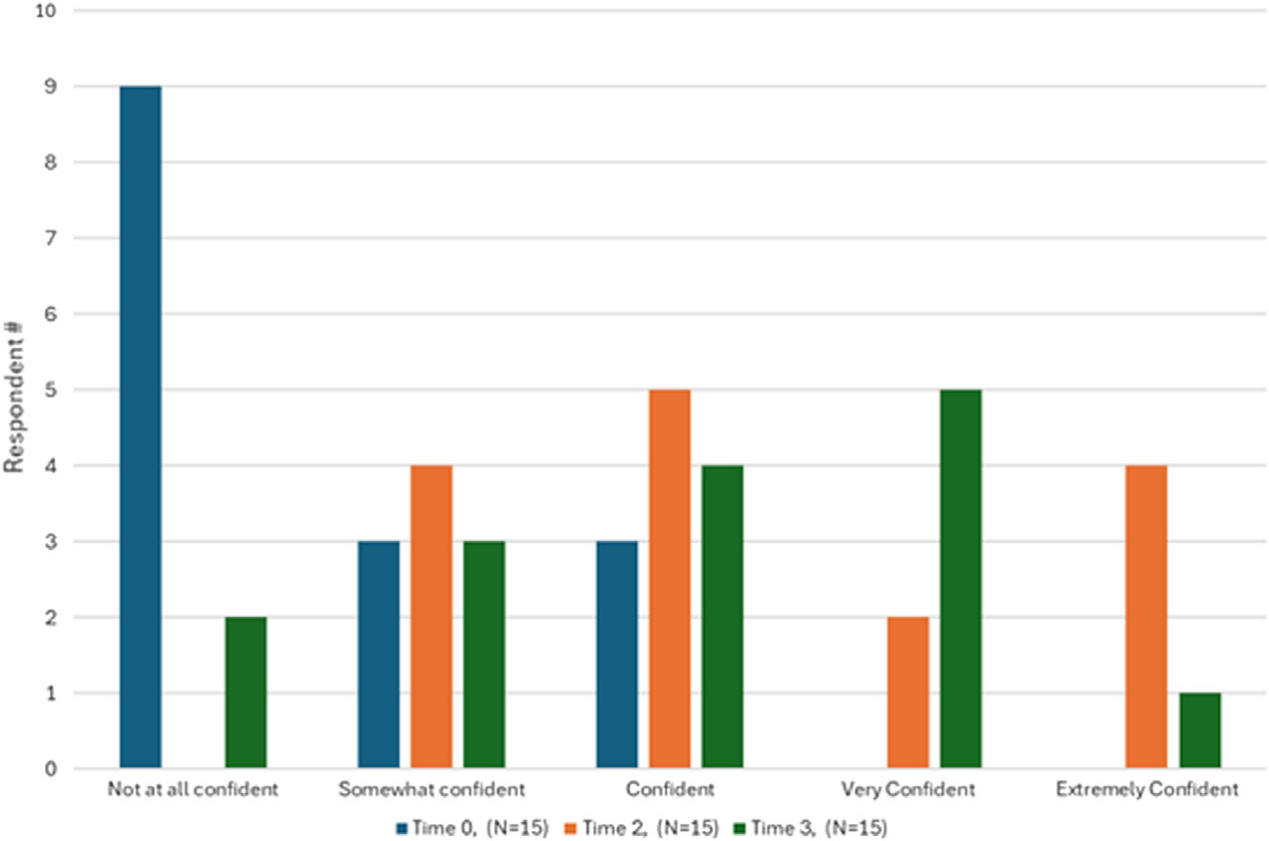
Learner confidence in teaching the slit-lamp exam at Time 0 (pre-curricular), Time 2 (immediate post-SBML curriculum), Time 3 (2-months post-SBML curriculum).

**Figure 4.**
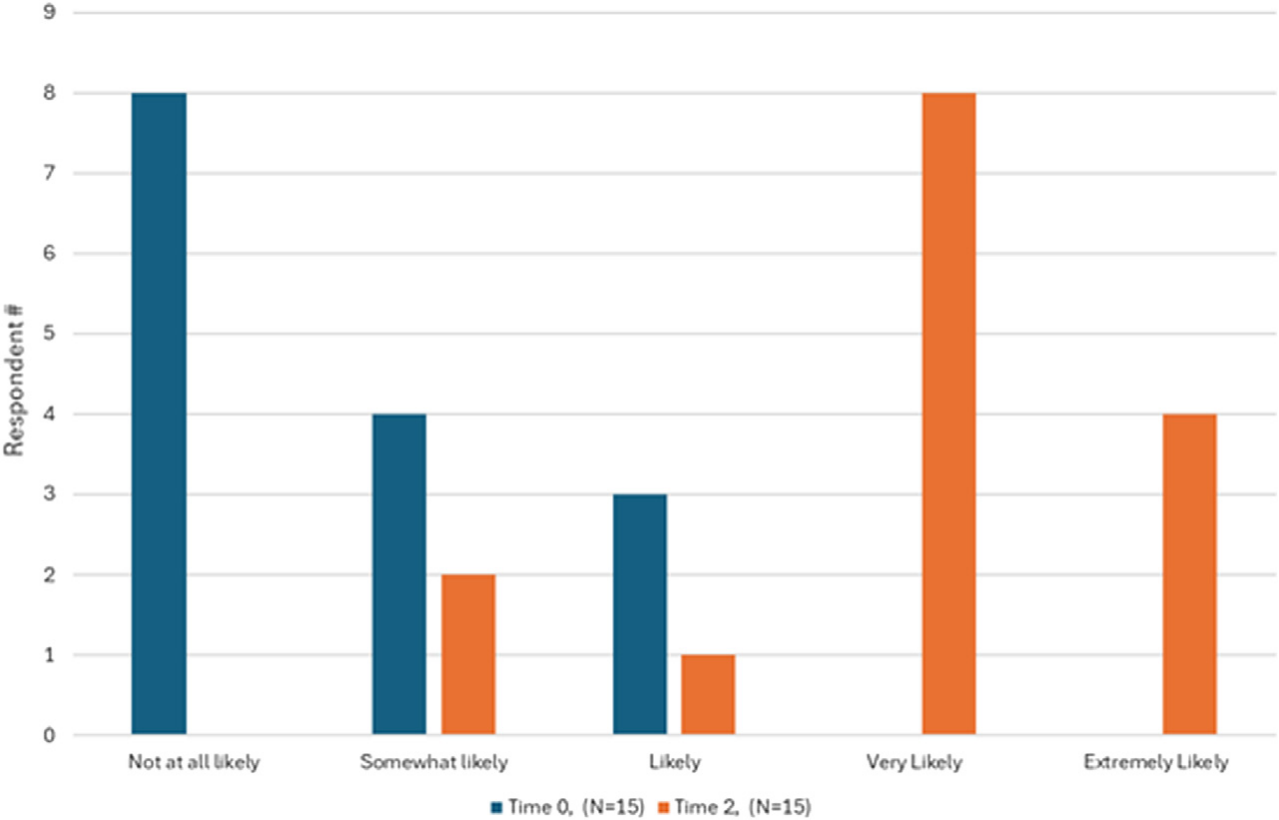
Learner likelihood in teaching the slit-lamp exam at Time 0 (pre-curriculuar) and Time 2 (immediate post-SBML curriculum).

Figures 2 and 3 illustrate learners’ confidence in performing and teaching the slit-lamp exam at the beginning of the study (Time 0), immediately after achieving procedural mastery (Time 2), and two months later (Time 3). Figure 4 illustrates the learners’ likelihood in teaching the slit-lamp exam at Time 0 and Time 2. Before participating in the slit-lamp curriculum, 73% of EPs also reported rarely or never performing a slit-lamp exam, while 80% of EPs reported sometimes or often using a Wood’s lamp for ocular complaints. Only 20% of EPs reported feeling confident in performing and teaching a comprehensive slit-lamp exam, while 67% of EPs reported feeling confident in using and teaching Wood’s lamp for ocular examination.

After completing the slit-lamp curriculum (Time 2), 86.7% of EPs reported feeling confident performing a comprehensive slit-lamp exam for ocular complaints, and 73.3% were more confident in teaching residents how to perform a slit-lamp exam. Most EPs strongly agreed that the ED SLIT LAMP curriculum helped them perform an independent slit-lamp exam and identify critical findings for common ocular complaints (80%), enhancing their learning more than traditional lectures and reading alone (86.7%). Of the asynchronous materials, the video demonstration was the most used (53% used it “a lot” or a “great deal”); the PowerPoint lecture and WEH Manual were the least used. At two months post-ED SLIT LAMP (Time 3), 73% and 67% of participants expressed extreme confidence in performing and teaching a resident how to perform a slit-lamp exam. Five t of 15 EPs reported teaching learners within the two-month post-curricular period, ranging from 5–30 students per EP participant.

Table 4 summarizes the statistically significant findings from the survey responses based on the three timeframes. There was a statistically significant increase in self-reported confidence in 1) performing a comprehensive slit lamp exam and 2) teaching residents to perform this exam between Time 0 to Time 2 and Time 0 to Time 3 (*P* < 0.001). There was no difference in reliance on ophthalmology consultation to modify or reinforce a treatment plan for ocular complaints when comparing Time 0 to Time 3 (*P* = 0.70, *P* = 0.814). There was also no statistical difference in the number of patients with ocular complaints evaluated by the study participants at the TJUH ED and WEH ED throughout the study (*P* = 0.14, *P* = 1.00).

**Table 3.** Statistical analysis of survey questions between the three different study timeframes.

Survey question	Time 0 median [IQR]^a^	Time 2 median [IQR]^b^	Time 3 median [IQR]^c^	Bonferroni adjusted *P*-value from Wilcoxon signed rank test time 0 vs. time 2	Bonferroni adjusted *P*-value from Wilcoxon signed rank test time 0 vs. time 3
Slit lamp					
Based on your current practice patterns: how confident are you in: performing a comprehensive slit lamp exam for ocular complaints?	1 [1, 2]	4 [3, 4.5]	3 [2.5, 4]	<0.001	<0.001
Based on your current practice patterns, how confident are you in: teaching residents to perform a comprehensive slit lamp exam for ocular complaints	1 [1, 2]	3 [2.5, 4.5]	3 [2, 4]	<0.001	0.004
How often do you: perform an independent slit lamp exam for ocular complaints?	2 [1, 2.5]	n/a^*^	3 [3, 3]	n/a^*^	0.064
Wood’s lamp					
Based on your current practice patterns, how confident are you in: performing a comprehensive Wood’s lamp exam for ocular complaints?	4 [2, 4]	4 [4, 5]	4 [3, 5]	0.016	0.03
Based on your current practice patterns, how confident are you in: teaching residents to perform a comprehensive Wood’s lamp exam (with access to a slit lamp) for ocular complaints?	4 [2, 4]	4 [4, 5]	4 [3, 5]	0.03	0.08
How often do you: use a wood lamp (with access to a slit lamp) for ocular complaints?	3 [3, 4]	n/a^*^	3 [3, 3]	n/a^*^	1.00
Ophthalmology consultation habits					
How confident are you in identifying common ocular pathology seen in your main work site (CC, MHD, Urgent Care)?	2 [2, 3]	n/a^*^	3 [3, 4]	n/a^*^	0.018
On average, how many eye pathologies do you see at the main work site?	10 [4, 15]	n/a^*^	5 [3, 12.5]	n/a^*^	0.14
On average, how many eye pathologies do you see at other facilities?	12 [0, 40]	n/a^*^	37.5 [13.5, 50]	n/a^*^	1.00
How often do you rely on ophthalmology consultation to: help modify your treatment plan for ocular complaints?	3 [3, 3]	n/a^*^	3 [2.5, 3]	n/a^*^	0.70
How often do you rely on ophthalmology consultation to: reinforce your treatment and plan for ocular complaints?	3 [2, 3]	n/a^*^	3 [2, 3]	n/a^*^	0.814
How often do you rely on ophthalmology consultation to: provide additional information and guidance to your treatment and plan for ocular complaints?	3 [3, 4]	n/a^*^	3 [3, 3.5]	n/a^*^	1.00

*Confidence levels: 1 = Not at all confident, 5 = Extremely confident*

*Frequency levels: 1 = Never, 5 = Always*

a Time 0 = pre-curricular evaluation.

b Time 2 = immediate post SBML exam. Frequency of slit lamp and Wood’s lamp use were intentionally omitted for Time 2 due to the close proximity between Time 0 and Time 2, thus resulting in ‘n/a’ for some calculations.

c Time 3 = three months after SBML exam.

*CC*, Jefferson Hospital in Center City Philadelphia; *MHD*, Jefferson Methodist Hospital; *IQR*, interquartile range.

## DISCUSSION

The ED SLIT LAMP curriculum allowed EPs to increase their use and confidence in performing slit-lamp exams in the ED. The impetus for the project arose from EPs’ intrinsic motivation to provide better patient care. Our participant population consisted primarily of junior faculty who were initially uncomfortable performing or teaching slit-lamp exams and preferred using the Wood’s lamp. Upon completing the curriculum, the EPs noted a significant increase in self-reported confidence in using slit-lamps and were teaching multiple junior learners during their study enrollment.

The improvement between the pre-and post-curricular procedural competency also demonstrates the importance of understanding the technical nuances of the slit-lamp exam and practicing critical device movement, such as careful patient positioning, adjusting of the chin straps, changing the microscope angulation, and adjusting varying slit-lamp beam lengths and widths for diagnosing a wide range of anterior ophthalmic pathologies. These skills are drastically different than those required to operate a Wood’s lamp, which acts primarily as a magnifying glass with UV capabilities.

Our curriculum achieved three of the four Kirkpatrick goals. The majority of the participants (over 80%) reported positive reaction to the curriculum (the curriculum helped them perform a slit-lamp exam, evaluate for common pathologies, and offered more than traditional lectures) (Level 1); all of the participants demonstrated procedural mastery at Time 2 (Level 2); upwards of 50 learners received instructions from the study participants on how to use the slit lamp at Time 3 (Level 3). While the reliance on ophthalmology consultation did not reveal statistically significant changes, we posit that improved procedural acumen resulted in more targeted consultation questioning and improved rapport between the medical disciplines.

Since our participants were board-certified EPs with limited availabilities, the most valued component of the curriculum was the in-person RCDP session with the ophthalmologist (Time 2). This was reflected in almost every CIQ item, with specific mention of direct guidance in positioning the beam to look for cells and flare. The most surprising element to many participants was how many ocular diagnoses required the slit-lamp exam and that learning the procedure was not as complicated as they had initially anticipated. In contrast, many of the participants felt most distanced or removed from the curriculum in reviewing the asynchronous learning materials.

We were unsurprised to see the confidence levels in using Wood’s lamp unchanged between the three different time frames. While the slit lamp offers a superior and in-depth evaluation of the anterior segment of the eye, we acknowledge that a comprehensive slit-lamp exam is time- and resource-consuming and may not affect the clinician’s management if the suspected pathology involves larger lesions, foreign bodies, or specific reaction to fluorescein staining. The Wood’s lamp remains an easier and more portable diagnostic tool for some ocular pathologies, and its use in the clinical arena is still acceptable in certain situations.

## LIMITATIONS

This study was conducted at a single, large, tertiary academic center with an affiliated ophthalmology hospital and supported with internal grant funding. While the results were positive, multiple factors ciykd prevent this study from being replicated, especially at community sites without a close relationship with ophthalmology. One of the most significant challenges is scheduling in-person evaluations in the pre-curricular session, as well as the final in-person training and examination. We encountered significant logistical challenges in creating a schedule that was amenable to the ophthalmologists, EPs (with unpredictable shift schedules), and research investigators, as well as finding a consistent space in the WEH and WEH ED that had access to an attached-observer scope to ensure the participants were focusing on the correct anatomic structure during their procedural demonstration. This was further exacerbated when accounting for the “unlimited attempts” for RCDP. As this was our pilot study with advanced learners, we over-budgeted a two-hour template for each learner, which drastically limited the number of participants we could schedule for the final in-person exam.

Due to the longitudinal nature of this study and several in-person components, maintaining participant recruitment and engagement was also difficult. Of the 50 eligible board-certified TJUH EPs, only 15 EPs volunteered to participate. The primary deterrence, when discussed with non-participants, was time restraints and commuting into the city for in-person evaluations and examinations. We suggest implementing dedicated teaching days (ie, conference days or faculty meetings) for larger participant recruitment and subsequent follow-up and examination.

This study was funded by an internal grant that provided minor financial incentives for the participants and standardized patient volunteers. While our needs-based analysis revealed participants were more focused on promoting better patient care, many of the participants expressed appreciation for the staggered gift cards, which also incentivized them to complete each timeline-specific survey. All other investigators’ efforts, in contrast, were in-kind and required dedicated non-academic and non-clinical time to enroll participants, record all the interactions, and provide unrestricted time availabilities for the final mastery assessment. This study was also unanimously supported by both departmental leaderships to promote a better collegial relationship and interdisciplinary education opportunity between organizations with the two principal investigators holding unique leadership positions, ophthalmology consulting director [CC] and EM clerkship director [XCZ]. We suspect that also positively affected our recruitment process and the success of this interdisciplinary training curriculum. As this study was conducted at an academic hospital in an urban setting, it has been suggested that academic centers likely overestimate EP comfort and confidence in the diagnosis and management of ophthalmic emergencies.^9^ Furthermore, the proximity between both EDs may skew the data, as these EPs are likely exposed to fewer ophthalmic emergencies than hospitals without a nearby eye-focused ED.

Ultimately, the biggest limitation to this pilot study was the lack of in-person skill assessment at the 60-day follow-up due to limited staffing and scheduling challenges. In lieu of an objective competency score, we leveraged self-reported confidence at the 60-day mark as an approximate measurement of the skill retention. We recognize that learners are poor at gauging their own abilities, both over- and underestimating their skills based on a variety of factors. It is notable that 80% of our learners were initially “not confident” in completing a comprehensive slit-lamp exam prior to the SBML curriculum and scored an average checklist score of 60%. At Time 2, almost 87% of responders were “confident” in completing a comprehensive slit-lamp exam after receiving an average checklist score of 95%. Unfortunately, there is no association between learners’ confidence and passing rate (score >18) at Time 0 (Pearson chi-square 3.46, *P* = 0.17) and Time 2 (Pearson chi-square 0.833, *P* = 0.66), respectively. While we are unable to predict how these learners would have performed on their slit-lamp exam test at day 60, we are encouraged to see the number of study participants who continued to teach slit-lamp exam for junior learners. We posit these participants will likely have improved sustained competence and decreased skill decay by actively teaching others. Future studies should be considered to add a final examination (procedure or multiple-choice question) to validate our results.

## CONCLUSION

Emergency physicians are expected to diagnose and manage ocular complaints as part of their training and clinical practice. Our primary focus was to create a rigorous methodologic training curriculum (slit-lamp exam) for a specialty-focused skillset that could result in downstream teaching. This project highlighted a significant need for slit-lamp exam training within our institution that led to a successful transdisciplinary simulation-based mastery learning curriculum and improved our EPs’ confidence in performing and teaching slit-lamp exams to future clinicians. Furthermore, this study demonstrates that adult learners, especially attending physician value direct interaction with clinical instructors when learning a new skillset and are intrinsically motivated to hone their skillset and teach it to future learners when they have achieved this mastery. We encourage other institutions to leverage SBML as a teaching modality for procedural-based training and advocate cross-discipline education initiatives. Future investigation could include creating a multicenter study to implement this curriculum at other academic institutions and potentially include it in EM residency training.

## Supplementary Information





