# Supplementary material for: Emergency Department Slit Lamp Interdisciplinary Training Via Longitudinal Assessment in Medical Practice
Source: West J Emerg Med. 2024 Aug 16;25(5):725–34. doi: 10.5811/westjem.18514 (PMC11418879; doi:10.5811/westjem.18514)
Supplement: Supplementary file 1 [file wjem-25-725-s001.docx]

**Appendix 1 - Independent Readiness Assessment Test (IRAT)**

**PART I: Slit Lamp Technical Pre-test (HIGHLIGHTED SECTIONS ARE ANSWERS)**

1. **Identify Slit-lamp Structures by placing the label number that correctly identifies the prompt**


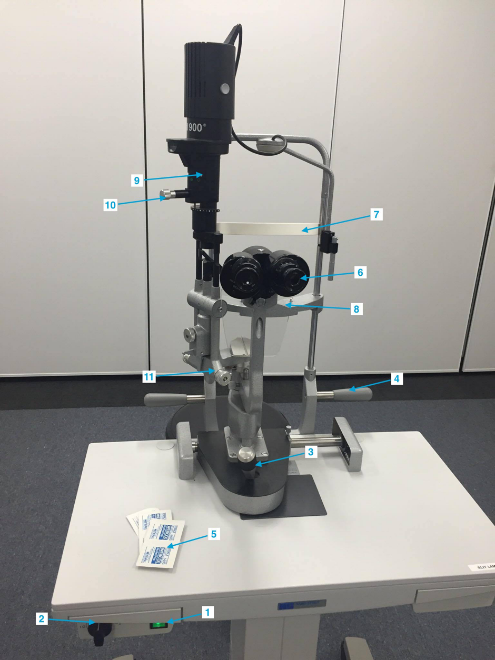


- 1. Slit beam length knob __10___
  2. Forehead Rest __7___
  3. Filter Changing Knob __9___
  4. On/Off switch __1___
  5. Eyepieces__6___
  6. Joystick___3__
  7. Chin rest__8___
  8. Slit beam width knob _11___
  9. Illumination __2___
  10. Handles for patient __4___

1. **Choose the first step from the list:**
   1. Instruct the patient to close his/her/their eyes
   2. Adjust the table height and chairs
   3. Sanitize the forehead and chin rest
   4. Adjust the eyepieces for your interpupillary distance and refractive error
2. **Circle True or False: Adjust the chin rest to align patient’s lateral canthus with black level (height) marker ring (below the forehead rest)**
   1. TRUE
   2. FALSE
3. **Choose the correct adjustment with the desired height of the light beam**
   1. Rotate the side joystick clockwise and counterclockwise
   2. Move the base joystick sideways for fine adjustments
   3. Move the joystick forward and backward for coarse adjustments
   4. Rotate the power supply clockwise and counterclockwise
4. **Circle True or False: on initial exam, magnification should be set on low power (10x to 12x), illumination at largest aperture, widest slit beam**
   1. TRUE
   2. FALSE
5. **Circle True or False: Turn on the light source by locating the box under the table with the rotary switch at the highest voltage setting**
   1. TRUE
   2. FALSE
6. **To check anterior chamber depth, ADJUST slit beam to a thin beam to and focus at which position on limbus?**
   1. 12:00
   2. 6:00
   3. 4:00
   4. 9:00
7. **Circle True or False: The cobalt blue filter should be used for fluorescein evaluation**
   1. TRUE
   2. FALSE
8. **Which anatomic landmark should you apply fluorescein?**
   1. Inferior fornix
   2. Superior sclera
   3. Medial lacrimal duct
   4. Anterior uvea
9. **How should you adjust the magnification, height, and width of your light beam to best see anterior cells and flare?**
   1. Low magnification, tall & wide
   2. Low magnification, short and wide
   3. High magnification, tall & thin
   4. High magnification, short and thin
10. **In what order would you assess for corneal abrasion?**
    1. Examine with blue light → instill fluorescein → Instill proparacaine → examine at slit lamp with white light
    2. Instill fluorescein → Instill proparacaine → examine at slit lamp with white light → examine with blue light
    3. Instill proparacaine → examine at slit lamp with white light → instill fluorescein --> examine with blue light
    4. Instill proparacaine → instill fluorescein → examine at slit lamp with white light → examine with blue light
11. **If you have trouble focusing, what are the likely causes?**
    1. Improper patient head position
    2. Oculars misaligned or set incorrectly
    3. A&B are both correct
    4. None of the above

**PART II: Clinical Image Examination (HIGHLIGHTED SECTIONS ARE ANSWERS)**

Quiz contents:

- Blepharitis
- Glaucoma
- Hyphema
- Cells and flares
- Synechia
- Hypopyon
- Lens dislocation
- Globe rupture
- Pseudodentrite/dentrite
- Follicular conjunctivitis
- Stye
- Perilimbal flush
- Corneal ulcer
- Corneal abrasion
- Keratoconjunctivitis Sicca
- Cataract

Image sources: courtesy from study investigator [CC] and from Wikimedia/Wikipedia

**What are the following diagnoses?**

1.
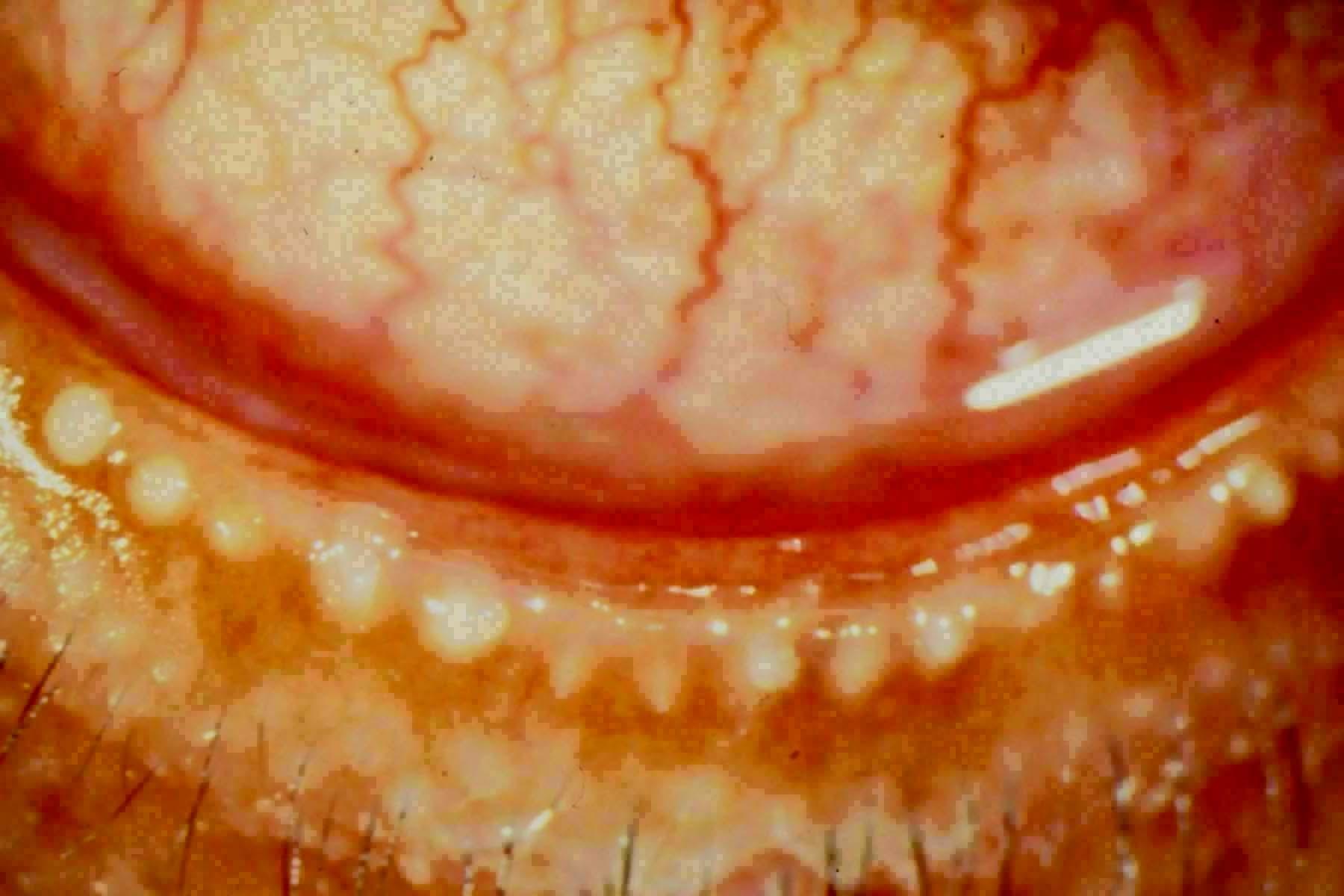

   1. Blepharitis
   2. Conjunctivitis
   3. Stye
   4. Synechia
2.
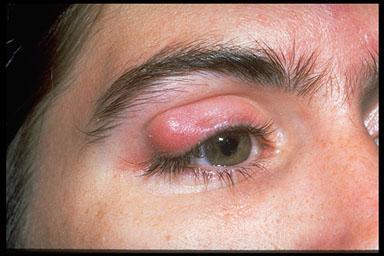

   1. Blepharitis
   2. Chalazion
   3. Dacrocystitis
   4. Periorbital cellulitis
3.
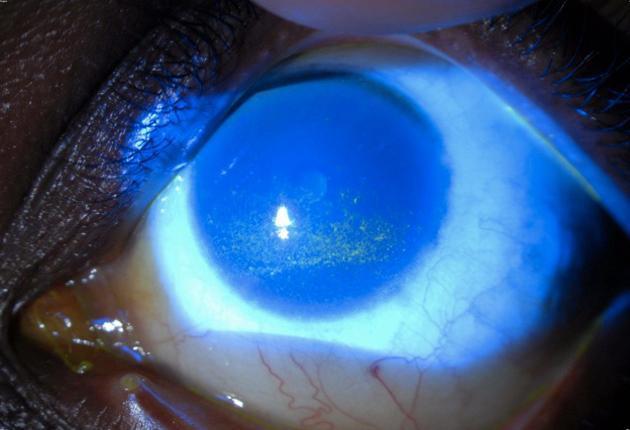

   1. Corneal abrasion
   2. Glaucoma
   3. Herpes ophthalmicus
   4. Keratoconjunctivitis sicca
4.
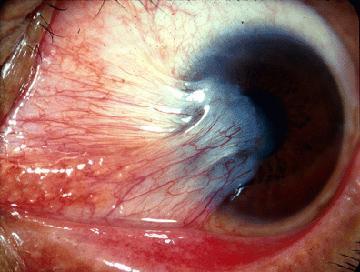

   1. Arterio-venous malformation
   2. Pinguecula
   3. Pterygium
   4. Subconjunctival hemorrhage
5.
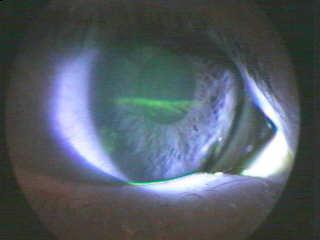

   1. Conjunctival abrasion
   2. Corneal foreign body
   3. Globe rupture
   4. Hypopyon
6.
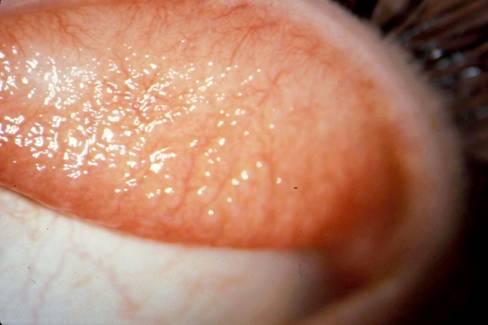

   1. Follicular conjunctivitis
   2. Keratoprecipitate
   3. Subconjunctival hemorrhage
   4. Uveitis
7.
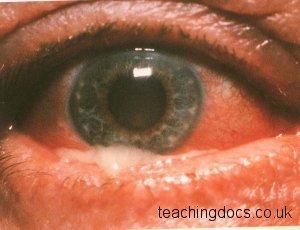

   1. Bacterial conjunctivitis
   2. Hyphema
   3. Stye
   4. Uveitis
8.
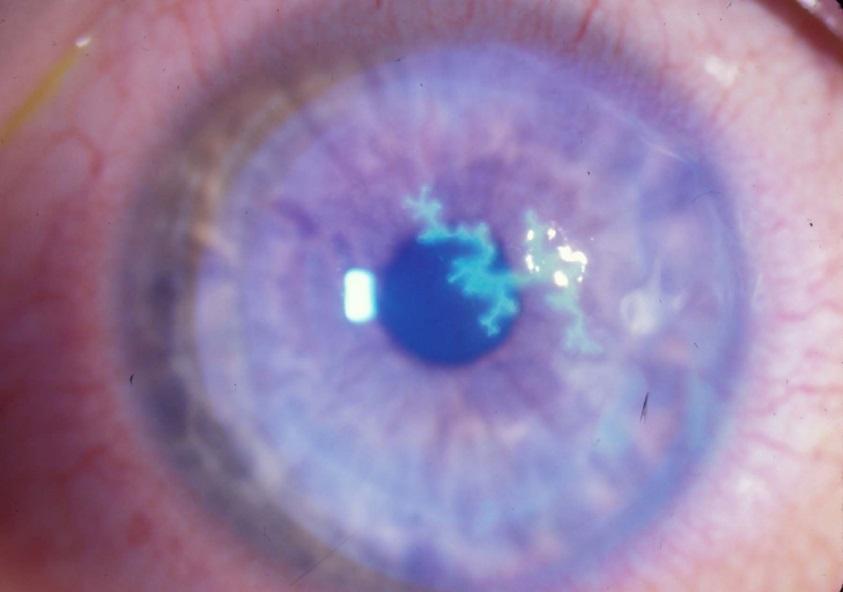

   1. Cataract
   2. Corneal abrasion
   3. Herpes keratitis
   4. Keratoprecipitate
9.
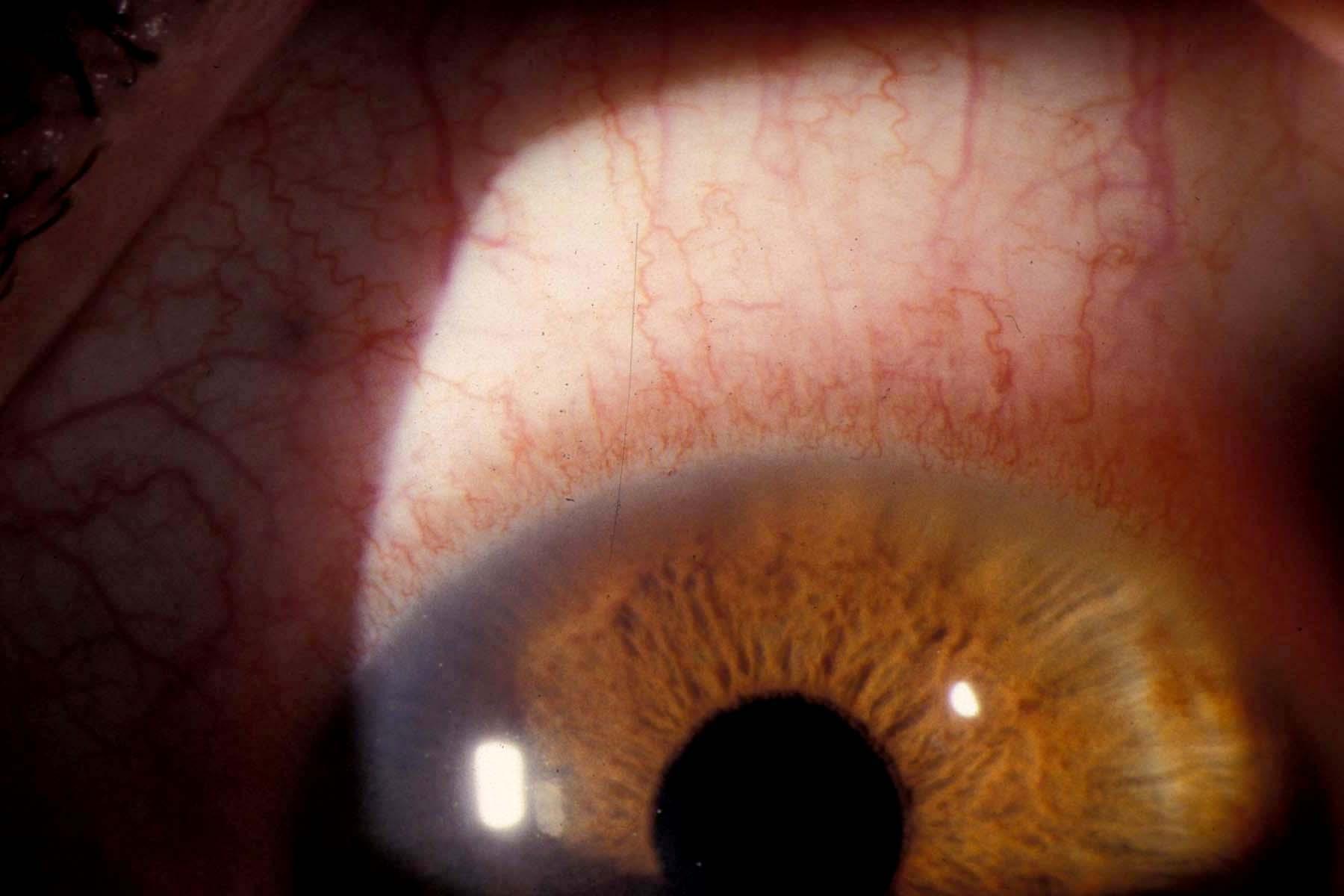

   1. Ciliary flush
   2. Conjunctivitis
   3. Subconjunctival hemorrhage
   4. Synechia
10.
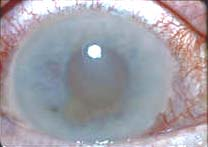

    1. Acute angle glaucoma
    2. Endophalmitis
    3. Globe rupture
    4. Uveitis
11.
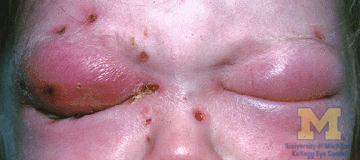
 https://commons.wikimedia.org/wiki/File:Orbital_cellulitis.jpg
    1. Blepharitis
    2. Erysipelas
    3. Orbital cellulitis
    4. Stye
12.
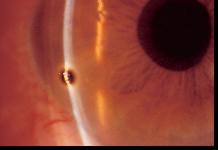

    1. Cells and flares
    2. Chemical burn
    3. Corneal foreign body
    4. Corneal melanoma
13.
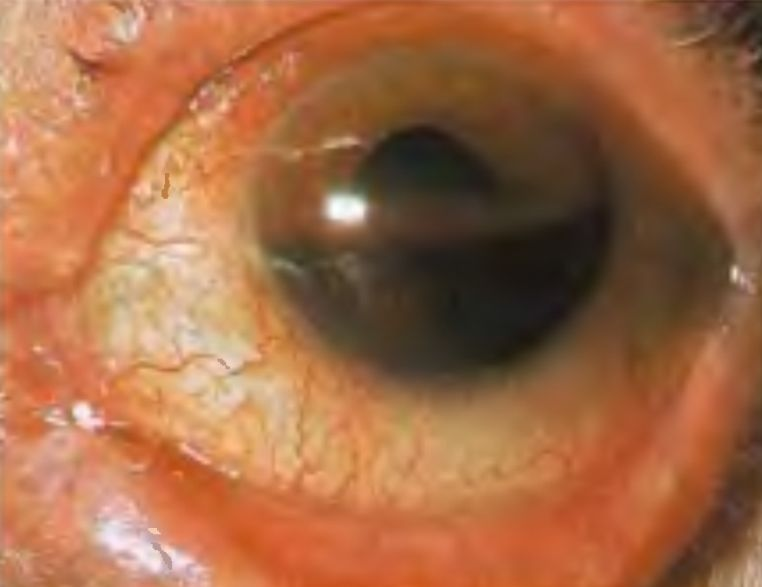


https://commons.wikimedia.org/wiki/File:Hyphema_-_occupying_half_of_anterior_chamber_of_eye.jpg

- 1. Chemical injury
  2. Hyphema
  3. Lens dislocation
  4. Vitreous hemorrhage

1.
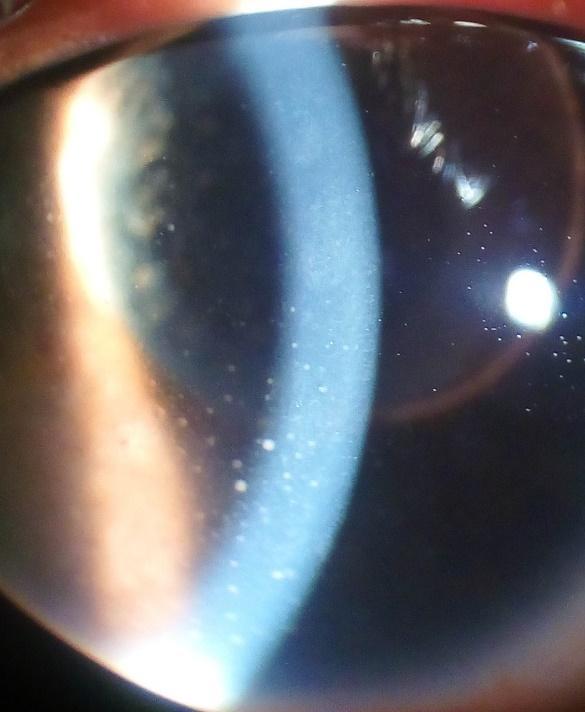
 https://en.wikipedia.org/wiki/Uveitis#/media/File:Keratic_precipitate2.jpg
   1. Cataracts
   2. Corneal laceration
   3. Lens dislocation
   4. Uveitis
2.
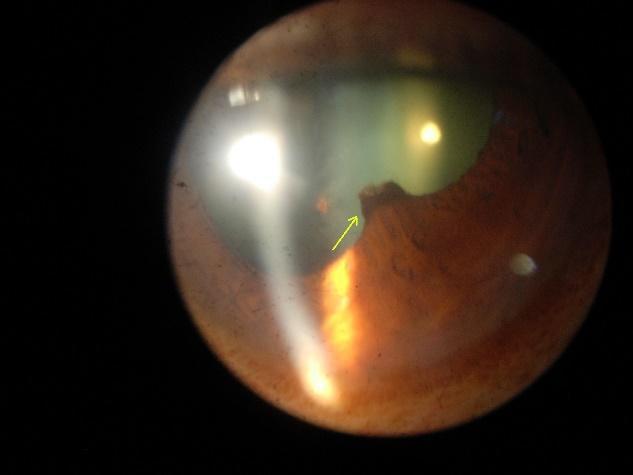
 https://en.wikipedia.org/wiki/Synechia_(eye)#/media/File:Posterior_synechia.jpg
   1. Glaucoma
   2. Irititis
   3. Squamous cell carcinoma
   4. Synechia
3.
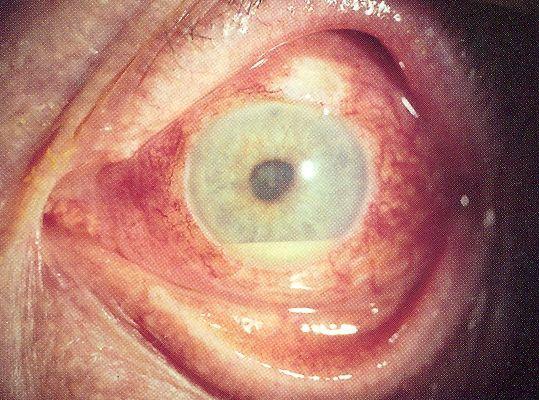
 https://en.wikipedia.org/wiki/Uveitis#/media/File:Hypopyon.jpg
   1. Acute angle glaucoma
   2. Globe rupture
   3. Hyphema
   4. Hypopyon
4.
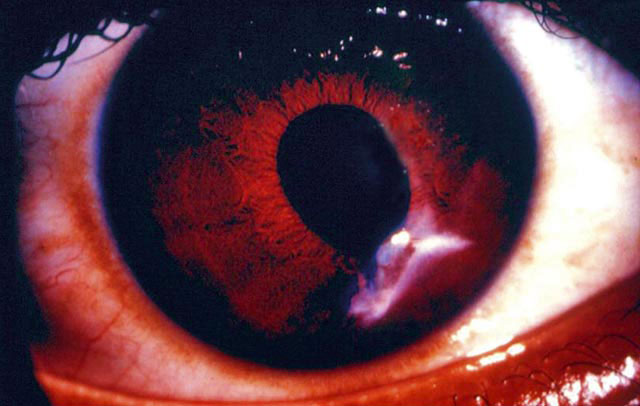

   1. Anterior uveitis
   2. Glaucoma
   3. Globe rupture
   4. Hypopyon
5.
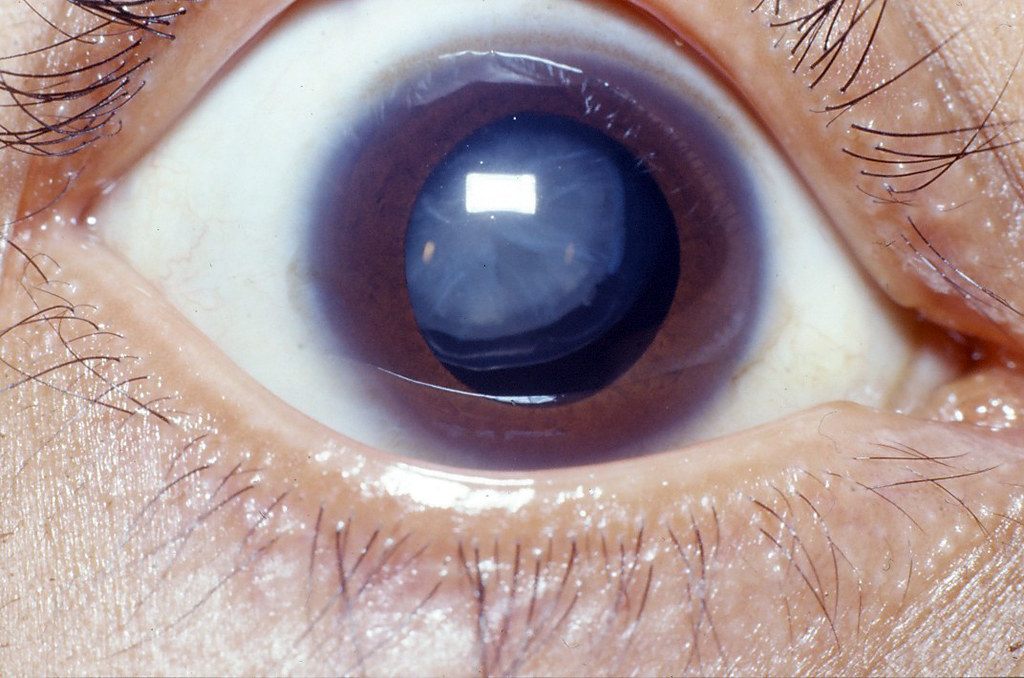
 https://www.flickr.com/photos/communityeyehealth/8411381000
   1. Anterior uveitis
   2. Glaucoma
   3. Globe rupture
   4. Lens subluxation
6.
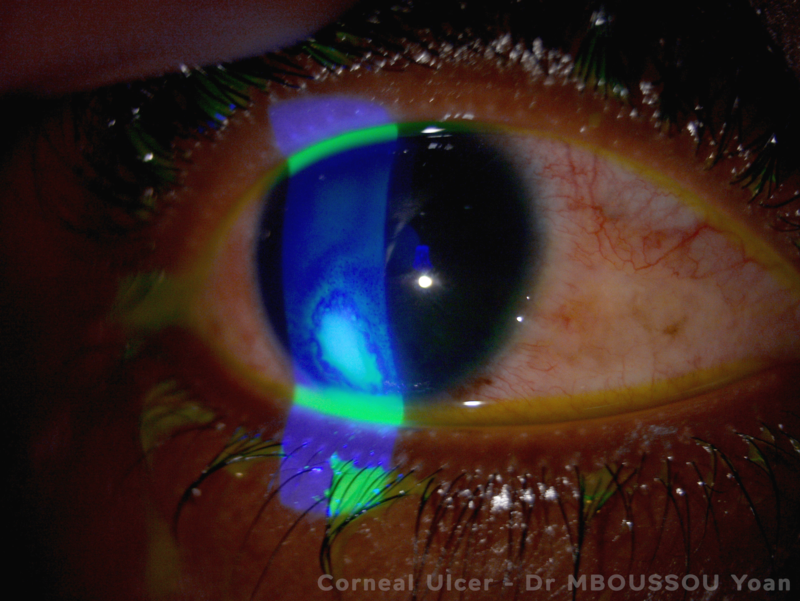
 https://en.wikipedia.org/wiki/Corneal_ulcer#/media/File:Corneal_Ulcer.png
   1. Corneal abrasion
   2. Corneal foreign body
   3. Corneal ulcer
   4. Hypopyon
7.
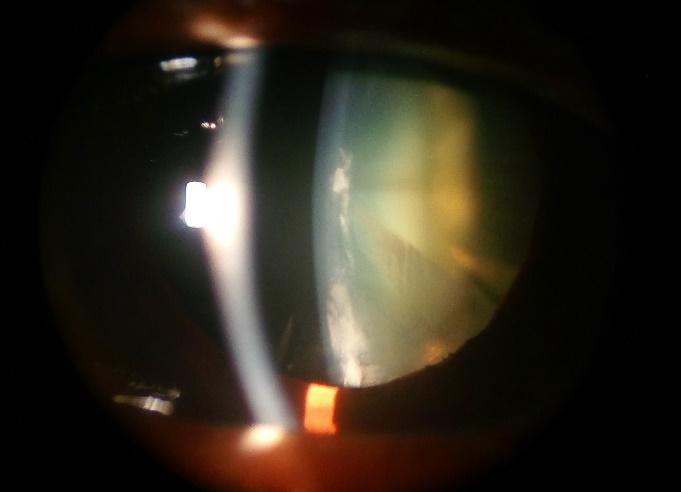
 https://commons.wikimedia.org/wiki/File:Cortical_Cataract.jpg
   1. Cataract
   2. Glaucoma
   3. Lens dislocation
   4. Ocular prosthesis

**PART III: Ophthalmology Exam Mix-n-Match (HIGHLIGHTED SECTIONS ARE ANSWERS)**

*Please select at least three (3) from the following list of clinical exam findings to describe the following diagnoses:*

**List of ocular findings:**

1. Lids swelling
2. Conjunctival injection
3. Eye discharge
4. Pseudomembrane
5. Palpebral follicular reaction
6. Cell and flare
7. Conjunctival injection
8. Kerotoprecipitate
9. Synechia
10. Cornea disruption
11. Anterior chamber [flat]
12. Iris abnormality
13. Sclera injection
14. Hypopyon
15. Hazy cornea
16. Non-reactive, mid-dilated pupil
17. Anterior chamber [shallow]
18. Meibomian gland inflammation
19. Lids/lashes debris
20. Superficial punctate keratitis
21. Dendrite
22. Pseudodentrite
23. Vesicles on lids
24. Follicular conjunctivitis

**List of Common Eye Pathologies w/Highlighted Findings (answers)**

**Conjunctivitis**

- Conjunctival injection
- Discharge
- Lids swelling
- Palpebral follicular reaction
- Pseudomembrane

**Uveitis**

- Cell and flare
- Conjunctival injection
- Keratoprecipitate
- Synechia

**Ruptured globe**

- Ant chamber flat
- Corneal disruption
- Injected
- Iris abnormality

**Endophthalmitis**

- Cells and flare
- Conjunctival injection
- Hazy cornea
- Hypopyon

**Angle-closure glaucoma**

- Conjunctival injection
- Corneal haze
- Non-reactive, mid-dilated pupil
- Anterior chamber [flat]

**Blepharitis**

- Lids/lashes debris
- Conjunctival injection
- Meibomian gland inflammation
- Superficial punctate keratitis

**Herpes simplex**

- Conjunctival injection
- Dendrite
- Follicular conjunctivitis
- Vesicles on lid
