# Supplementary material for: Emergency Department Slit Lamp Interdisciplinary Training Via Longitudinal Assessment in Medical Practice
Source: West J Emerg Med. 2024 Aug 16;25(5):725–34. doi: 10.5811/westjem.18514 (PMC11418879; doi:10.5811/westjem.18514)
Supplement: Supplementary file 2 [file wjem-25-725-s002.docx]

**Appendix 2 - Slit Lamp Final Checklist**

| Steps | Checkbox |
| --- | --- |
| **Step 1: Identifying slit lamp anatomy** |  |
| 1. On/off switch 2. Illumination 3. Joystick 4. Handles for patient 5. Alcohol swabs 6. Eyepieces 7. Forehead rest 8. Chin rest 9. Filter Changing Knob 10. Slit beam length knob 11. Slit beam width knob   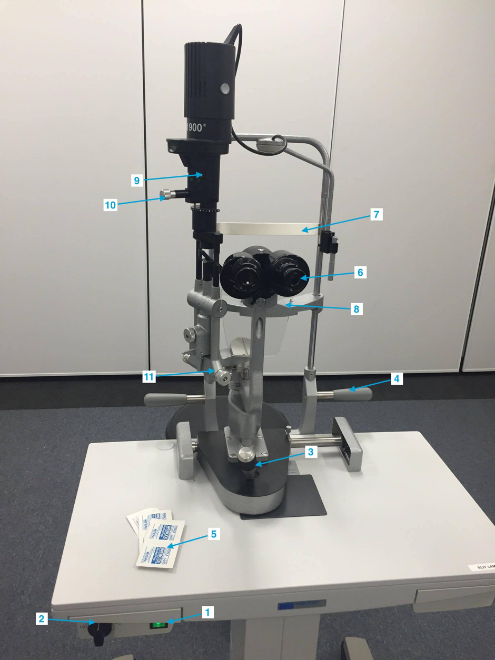 | 1 □ |
|  |  |
| **Step 2: Prepare instrument and patient** |  |
| - Apply transparent face shield over the slit lamp (COVID). | 2 □ |
| - Sanitize forehead and chin rest for the patient. | 3 □ |
| - Apply topical tetracaine/proparacaine on patient’s eyes. | 4 □ |
| - Unlock instrument base and shift by pulling toward you. | 5 □ |
| - Adjust eye pieces for your interpupillary distance and refractive error. | 6 □ |
| - Adjust table height and/or chair(s) - neither patient nor examiner should be hunched over. | 7 □ |
| - Instruct patient to close eyes while you power up by turning on the light source at low voltage setting and focus on right eyelid. Position patient in slit lamp with forehead touching the horizontal bar and chin in the chin rest. | 8 □ |
| - Set magnification on lowest settings (10x to 12x), illumination at largest aperture and widest slit beam. | 9 □ |
| - Adjust chinrest so the patient is sitting comfortably with their chin on the chinrest and their forehead against the headrest. | 10 □ |
|  |  |
| **Step 3: Illuminate ocular structures** |  |
| - Practice macro and micro adjustments of the sliding base with joystick. | 11 □ |
| - Adjust microscope 90° to facial plane with illumination set at 45° angle (angle LEFT for patient’s right eye, and RIGHT for left eye). | 12 □ |
| - Perform outer structure evaluation:   - The mirror should be slightly at an angle (more comfortable to the patient).   - Start laterally, look at the lids and the lashes and lacrimal apparatus while moving medially.   - Next, exam the conjunctiva, sclera and cornea.   Must complete for RIGHT EYE and LEFT EYE. | 13 □ |
| - Perform anterior chamber evaluation:   - Adjust the light beam to maximum height and minimum width   - Focus thin slit beam at 9:00 position on limbus. Move across the cornea to the 3:00 position by tilting joystick laterally.     1. For the RIGHT eye, move the mirror to your left at a 45° angle.     2. For the LEFT eye, move the mirror to your right at a 45° angle.   - Note the position of the curved corneal beam relative to the flat iris beam, and the space between the beams. Anterior chamber depth is wide if space between beams = corneal thickness just inside the limbus. A/C is shallow if space is < 1/4th cornea.   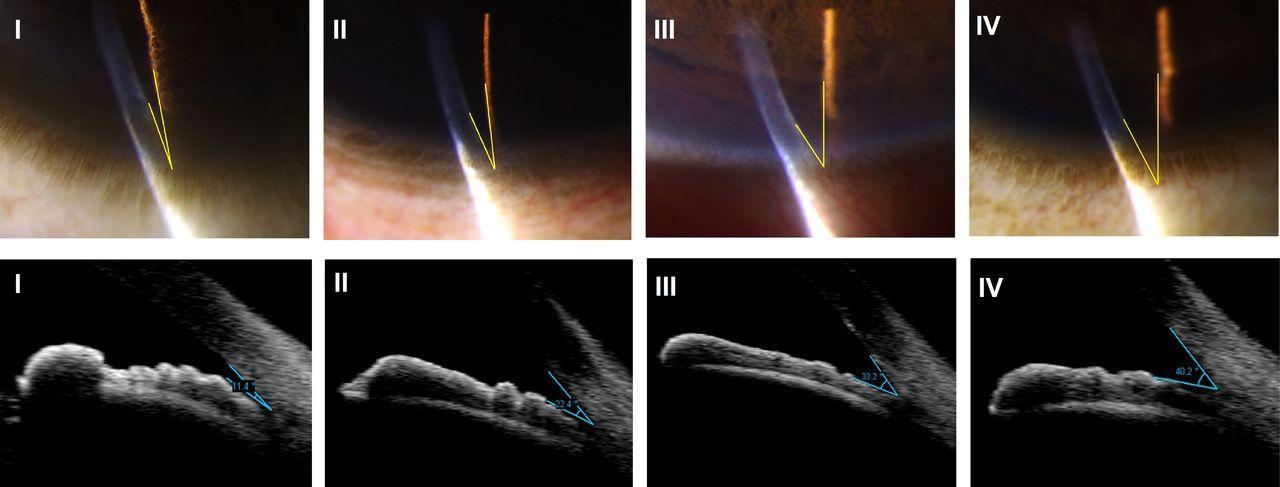  <https://bjo.bmj.com/content/103/7/960>  Must complete for RIGHT EYE and LEFT EYE | 14 □ |
| - Look for cells and flare   - Shorten the height of the beam to 3-4mm and keep beam as narrow as possible.   - Switch the magnification lever to the higher setting.   - Focus on the cornea, then slide the joystick forward slightly to focus on the anterior surface of the lens.   - Slowly slide the joystick backwards to focus on a point midway between the cornea and the anterior surface of the lens.   - Keep the beam centered over the pupil (the black background makes it easier to see cells and flare).   - Angle beam about 45 degrees   Must complete for RIGHT EYE and LEFT EYE | 15 □ |
| **Step 4: Perform fluorescein evaluation** |  |
| - Place a drop of tetracaine/proparacaine on a sterile fluorescein strip. | 16 □ |
| - The fluorescein is then placed in the inferior fornix of the eye by pulling down on the lower lid and gently touching the bulbar conjunctiva with the fluorescein strip. | 17 □ |
| - Adjust cobalt blue filter on diaphragm wheel at maximum beam height and medium width slit setting for fluorescein evaluation. | 18 □ |
| - Focus the slit beam at 9:00 position on limbus. Move across the cornea to the 3:00 position by tilting joystick laterally   Must complete for RIGHT EYE and LEFT EYE | 19 □ |
| **Step 5: Exam completion** |  |
| - Pull instrument base toward you when finished and lock in position. Turn off. | 20 □ |
