# Supplementary material for: Emergency Department Slit Lamp Interdisciplinary Training Via Longitudinal Assessment in Medical Practice
Source: West J Emerg Med. 2024 Aug 16;25(5):725–34. doi: 10.5811/westjem.18514 (PMC11418879; doi:10.5811/westjem.18514)
Supplement: Supplementary file 3 [file wjem-25-725-s003.docx]

**Appendix 5 - Slit Lamp Surveys**

**Time 0 - Pre-curricular survey**

**Q1-3: Demographics**

1. What is your gender? (select all that apply)
   1. Male
   2. Female
   3. Trans male/man
   4. Trans female/woman
   5. Genderqueer/Gender non-conforming
   6. Different Identity
   7. Do not wish to disclose
2. I identify myself as the following race/ethnicity. Please indicate all that apply.
   1. American Indian or Alaska Native
   2. Asian
   3. Black or African American
   4. Hispanic/Latino
   5. Native Hawaiian or Pacific Islander
   6. White
   7. Other
3. My age range is:
   1. 25-29 years old
   2. 30-34 years old
   3. 35-39 years old
   4. 40-44 years old
   5. 45+ years old
4. How long have you practiced EM post residency graduation?
5. 1-3 years
6. 4-6 years
7. 7-9 years
8. 10+ years

Please answer the following questions #5-9 to the best of your recollection:

Over the **past 3 months...**

1. On average, how many eye pathologies do you see at the main work site (CC, MHD, Urgent care)? (SLIDING SCALE 0-20)
2. On average, how many eye pathologies do you see at other facilities (i.e. Wills Eye), if applicable? (SLIDING SCALE 0-20)
3. On average, how often do you perform an independent slit lamp exam for ocular complaints? (SLIDING SCALE 0-20)
4. On average, how often do you use a wood lamp (with access to a slit lamp) to evaluate eye pathology for ocular complaints? (LIKERT)
5. On average, how many times do you rely on ophthalmology consultation to help:
   1. Modify your treatment and plan for ocular complaints?
   2. Reinforce your treatment and plan for ocular complaints?
   3. Provide additional information and guidance to your treatment and plan for ocular complaints?

The following questions refer to familiarity with the **slit lamp**…(LIKERT SCALE)

1. How confident are you in performing a comprehensive **slit lamp** exam for ocular complaints?
2. How confident are you in your ability to teach residents to perform a comprehensive **slit lamp** exam for ocular complaints?
3. How likely are you to teach learners to perform a comprehensive **slit lamp** exam for ocular complaints?

The following questions refer to familiarity with the **woods lamp** (WITH ACCESS TO A SLIT LAMP)…(LIKERT SCALE)

1. How confident are you in performing a comprehensive **woods lamp** exam for ocular complaints?
2. How confident are you in your ability to teach learners to perform a comprehensive **woods lamp** exam (with access to a slit lamp) for ocular complaints?
3. How likely are you to teach learners in performing a comprehensive **woods lamp** exam (with access to a slit lamp) for ocular complaints?
4. How confident are you in identifying common ocular pathology seen in your main work site (CC, MHD, Urgent care)?

**Time 2 - Post Mastery Checklist survey**

The following questions refer to familiarity with the **slit lamp**…(LIKERT SCALE)

1. How confident are you in performing a comprehensive **slit lamp** exam for ocular complaints?
2. How confident are you in your ability to teach residents to perform a comprehensive **slit lamp** exam for ocular complaints?
3. How likely are you to teach learners to perform a comprehensive **slit lamp** exam for ocular complaints?

The following questions refer to familiarity with the i.e., **woods lamp** (WITH ACCESS TO A SLIT LAMP)…(LIKERT SCALE)

1. How confident are you in performing a comprehensive **woods lamp** exam for ocular complaints?
2. How confident are you in your ability to teach learners to perform a comprehensive **woods lamp** exam (with access to a slit lamp) for ocular complaints?
3. How likely are you to teach learners in performing a comprehensive **woods lamp** exam (with access to a slit lamp) for ocular complaints?
4. How confident are you in identifying common ocular pathology seen in your main work site (CC, MHD, Urgent care)?

The following questions refer to the **ED SLIT LAMP mastery learning curriculum…** (LIKERT SCALE)

1. This curriculum helped you perform an independent, comprehensive slit lamp exam
2. The curriculum helped you evaluate for critical clinical findings for common ocular complaints presenting in your main work site
3. The knowledge from this curriculum would help during your future career
4. This curriculum enhanced learning more than traditional lectures and reading alone
5. **Which elements of the asynchronous ED SLIT LAMP learning materials did you most utilize, before your in-person session.** (LIKERT)
   1. Powerpoint
   2. Video demonstration
   3. Checklist
   4. Wills Manual
   5. Other__________

End of the course evaluation **(Critical Incident Questionnaire)**: (FILL IN THE BLANK)

1. At what moment during the activity did you feel most engaged with what was happening?
2. At what moment during the activity did you feel most distanced from what was happening?
3. What action did anyone (i.e., faculty member or peer) take during the activity that you found most affirming and helpful?
4. What action did anyone (i.e., faculty or peer) take during the activity that you found most puzzling or confusing?
5. What about the activity surprised you the most? (This could be something about your own reactions to what went on, or something that someone did, or anything else that occurs to you.)

**Time 3 - ED SLIT LAMP follow up survey**

Since completing the mastery learning curriculum (approximately 3 months ago)

1. On average, how many eye pathologies do you see at the main work site (CC, MHD, Urgent care)? (SLIDING SCALE 0-20)
2. On average, how many eye pathologies do you see at other facilities (i.e. Wills Eye), if applicable? (SLIDING SCALE 0-20)
3. On average, how often do you perform an independent slit lamp exam for ocular complaints? (SLIDING SCALE 0-20)
4. On average, how often do you use a wood lamp to evaluate eye pathology for ocular complaints? (LIKERT)
5. On average, how many times do you rely on ophthalmology consultation to help:
   1. Modify your treatment and plan for ocular complaints?
   2. Reinforce your treatment and plan for ocular complaints?
   3. Provide additional information and guidance to your treatment and plan for ocular complaints?
6. On average, how many eye pathologies do you see at the main work site (CC, MHD, Urgent care)? (SLIDING SCALE 0-20)
7. On average, how many eye pathologies do you see at other facilities (i.e. Wills Eye), if applicable? (SLIDING SCALE 0-20)
8. On average, how often do you perform an independent slit lamp exam for ocular complaints? (SLIDING SCALE 0-20)
9. On average, how often do you use a wood lamp (with access to a slit lamp) to evaluate eye pathology for ocular complaints? (LIKERT)
10. On average, how many times do you rely on ophthalmology consultation to help:
    1. Modify your treatment and plan for ocular complaints?
    2. Reinforce your treatment and plan for ocular complaints?
    3. Provide additional information and guidance to your treatment and plan for ocular complaints?

The following questions refer to familiarity with the **slit lamp**…(LIKERT SCALE)

After completing the mastery learning curriculum...

1. How confident are you in performing a comprehensive **slit lamp** exam for ocular complaints?
2. How confident are you in your ability to teach residents to perform a comprehensive **slit lamp** exam for ocular complaints?
3. How likely are you to teach learners to perform a comprehensive **slit lamp** exam for ocular complaints?

The following questions refer to familiarity with the i.e., **woods lamp** (WITH ACCESS TO A SLIT LAMP)…(LIKERT SCALE)

After completing the mastery learning curriculum...

1. How confident are you in performing a comprehensive **woods lamp** exam for ocular complaints?
2. How confident are you in your ability to teach learners to perform a comprehensive **woods lamp** exam (with access to a slit lamp) for ocular complaints?
3. How likely are you to teach learners in performing a comprehensive **woods lamp** exam (with access to a slit lamp) for ocular complaints?
4. How confident are you in identifying common ocular pathology seen in your main work site (CC, MHD, Urgent care)?

Since completing the mastery learning curriculum (approximately 3 months ago)…

1. Have you instructed other learners on how to use a slit lamp? If yes, please indicate the level of the learner (EP, resident, APP, medical student, PA student), and number of learners you have instructed.
2. How many patients have you treated or evaluated and dispositioned an eye complaint in the ED that you would have previously consulted ophthalmology for?
